# Do men face greater barriers to accessing HIV testing services than women? Might HIV self-testing be the answer? Evidence from a longitudinal survey in east Zimbabwe (2018–2023)

**DOI:** 10.1371/journal.pgph.0006125

**Published:** 2026-03-24

**Authors:** Wilfred Ouma Otambo, Phyllis Mandizvidza, Louisa Moorhouse, Maxime Inghels, Reuben Moyo, Elphas Okango, Blessing Tsenesa, Rufurwokuda Maswera, Freedom Dzamatira, Simon Gregson, Frank Tanser, Constance Nyamukapa, Paul Mee

**Affiliations:** 1 South African Centre for Epidemiological Modelling and Analysis (SACEMA), Centre for Epidemic Response and Innovation (CERI), School for Data Science and Computational Thinking, Stellenbosch University, Cape Town, South Africa; 2 Biomedical Research and Training Institute, Harare, Zimbabwe; 3 MRC Centre for Global Infectious Disease Analysis, School of Public Health, Imperial College London, London, United Kingdom; 4 Lincoln Institute for Rural and Coastal Health, College of Health and Science, University of Lincoln, Lincoln, United Kingdom; 5 Africa Health Research Institute, KwaZulu-Natal, South Africa; PLOS: Public Library of Science, UNITED STATES OF AMERICA

## Abstract

HIV testing is crucial for prevention and treatment, yet barriers may disproportionately affect men in some sub-Saharan African settings. HIV self-testing (HIVST) may offer a promising solution, but its uptake remains under-explored. This study examines sex-specific barriers, HIV testing trends, and HIVST uptake in Manicaland, Zimbabwe, across three survey rounds pre-, during-, and post-COVID-19. We conducted longitudinal surveys 2018–2019 (pre- COVID-19), 2021 (during COVID-19), 2022–2023 (post-COVID-19), across six socio-economic zones (tea estates, rural villages, forestry areas, towns, roadside settlements, and urban areas) in eastern Zimbabwe. The study employed an open cohort design with repeated cross-sections, complemented by a sub-analysis among repeat participants to assess robustness. Data on socio-demographic characteristics, HIV testing, perceived barriers, and HIVST awareness and usage were analysed using generalized linear mixed-effects models. Men consistently had lower odds of HIV testing than women (2022–2023, aOR=0.74, 95% CI:0.67–0.81, *P* < 0.001). Barriers to HIV testing peaked during COVID-19 (2021), with men reporting three to five times higher barriers than women, including “judgmental staff” (24.5% vs. 4.7%, *P* = 0.005) and “lack of privacy” (21.5% vs. 5.1%, *P* = 0.032). Rural residents had reduced testing odds post-COVID-19 (aOR=0.82, 95% CI:0.69–0.99, *P* = 0.034). Although HIVST awareness increased, particularly among men compared to women, (66.7% vs 58.5%, aOR=2.21, 95% CI:2.09–2.33, *P* < 0.001), usage remained low (5.9%, vs 9.8%). Men report substantially greater barriers to HIV testing in Zimbabwe, exacerbated during the COVID-19 and are now further threatened by cuts to HIV funding. Although HIVST awareness expanded, its low uptake among men highlights the need for improved linkage-to-care mechanisms and tailored outreach such as peer-led distribution models.

## Introduction

The global response to HIV has achieved significant progress in prevention, treatment, and care, with substantial reductions in new infections and improved access to antiretroviral therapy [1,2]. Despite these advancements, HIV remains a major public health challenge in sub-Saharan Africa, which accounts for 25.6 million of the estimated 39.0 million people living with HIV globally in 2023 [3]. Within this region, Zimbabwe is a high-burden country with a national HIV prevalence of 12.9% and 1.3 million people living with HIV [4]. In Manicaland Province in Zimbabwe, the prevalence is slightly lower at 10.2%, yet the region continues to face significant challenges in controlling the epidemic [5].

HIV testing is a key component of the HIV response, enabling early diagnosis, timely initiation of treatment, and effective prevention strategies and behavior change interventions [6,7]. Despite financial incentives acting as a motivator in increasing testing uptake among men [8,9], equitable access in resource-limited settings remains a critical barrier [10–12]. Globally, testing coverage has improved, but disparities persist with men often testing less frequently than women, with uptake rates 10–20% lower in many sub-Saharan African countries [1,13–15]. Systematic reviews highlight social norms around masculinity, fear of stigma, and limited engagement with health services as key male-specific obstacles [10,16]. Before the COVID-19 pandemic, surveys documented lower testing rates among men compared to women, exacerbated by occupational demands and clinic hours misaligned with male work patterns [17,18]. During the COVID-19 pandemic, facility-based HIV testing in Zimbabwe declined and more pronounced among men [18]. The pandemic introduced interruptions in health service delivery, altering access patterns and exacerbating existing inequities [19,20]. Men, prioritizing economic recovery, were less likely to return for testing as services resumed [21,22]. While HIV testing rates began to rebound after the COVID-19 pandemic, recovery was uneven with community-based campaigns and targeted interventions primarily reaching women, especially in urban areas, while rural men remained underserved [18]. This has raised concerns about persistent sex gaps in HIV prevention and care [17,22,23]. Limited longitudinal data exist on barriers men face in accessing care pre-, during, and post-pandemic particularly in diverse sub-Saharan African contexts such as Zimbabwe.

HIV self-testing (HIVST) has emerged as a promising scalable strategy to bridge testing gaps in hard-to-reach groups including men [24–27]. Trials demonstrate that HIVST uptake is high compared to facility-based testing, with peer distribution enhancing reach [28,29]. However, linkage to confirmatory testing and care remains sub optimal, undermined by price sensitivity, and motivational barriers [30,31]. HIVST services adapted rapidly during the pandemic, leveraging online platforms, mail-out kits, and community-based distribution to maintain access when facility-based testing was disrupted enabling continued uptake [32–36]. Despite these successes, most studies either focus on specific sub-populations or use cross-sectional or short-term designs [25,26,28,37]. Emerging experimental evidence demonstrates the potential of innovative digital approaches such as chatbot-based intervention, to significantly increase HIVST uptake and engagement [38]. More robust, longitudinal data are needed to understand how different groups accessed and benefited from HIVST during and after the pandemic are needed. Furthermore, there is limited evidence on how awareness and uptake of HIVST have evolved, in response to disruptions caused by the COVID-19 pandemic.

This study analyses longitudinal data from Manicaland, Zimbabwe, to investigate differences in HIV testing barriers, explore shifts in testing behavior across three periods pre-COVID-19 (2018–2019), during-COVID-19 (2020–2021), and post-COVID-19 (2022–2023). The study identifies period-specific shifts in sex-stratified barriers, isolates COVID-19’s differential impact on testing behaviors, and tracks HIVST awareness and uptake trajectories. These analyses carry immediate implications validating peer-led, community-embedded HIVST as a pandemic-resilient strategy to re-engage men, directly informing the IMPERATIVE trial (ClincalTrials.gov NCT06370923), which leverages male peer networks to enhance HIVST and Pre-exposure prophylaxis (PrEP) uptake among men in Zimbabwe. This research aims to guide scalable, inclusive strategies for improving HIV testing and prevention in high-burden settings.

## Methods

### Ethics statement

Ethical approval for the study was obtained from both the Imperial College Research Ethics Committee (reference number 20IC6436) and the Medical Research Council of Zimbabwe (MRCZ/A/2703). Informed consent was obtained from all participants prior to their enrollment. For participants under the age of 18, written informed consent was secured from a parent or guardian, along with assent from the minor. Verbal consent procedures were used during telephone interviews, and these were audio-recorded with participant permission. All recordings were stored separately from the primary data to preserve confidentiality. Throughout the study, strict ethical standards were upheld to ensure the rights, safety, and dignity of all participants.

### Survey design and setting

This study utilized data from the Manicaland general population cohort study, a longitudinal open cohort consisting of repeated cross-sectional surveys conducted in Manicaland Province, eastern Zimbabwe, established in 1998 to monitor HIV epidemiology and assess the impact of public health interventions in the region [39]. The open cohort design with repeated cross-sections allows some participants to be followed longitudinally across survey rounds, while new participants were recruited in each round to maintain population representativeness allowing for analysis of both individual-level changes over time and population-level trends. In dynamic settings such as Manicaland, where migration, mortality, and loss to follow-up are substantial, repeated cross-sections reduce selection bias that can arise from conditioning only on long-term cohort retention. The design allows for comparison of population-level trends over time, with some participants appearing in multiple rounds and allows for inclusion of newly eligible participants and captures temporal shifts in testing behavior associated with structural changes, including COVID-19–related service disruptions. The present analysis draws on three rounds of data collection: Round 7 (July 2018 to December 2019), conducted prior to the COVID-19 pandemic; Round 8 (February to July 2021), carried out during the pandemic; and Round 9 (July 2022 to January 2023), conducted in the post-pandemic period. Each round was designed to be representative of the resident adult population at the time of data collection, with standardized sampling procedures and harmonized questionnaires across survey rounds.

The datasets were accessed for research purposes on 27 February 2024. The authors did not have access to information that could directly identify individual participants during and after data retrieval. These rounds were selected to examine changes in HIV testing behaviors, barriers to testing, and the uptake of HIV self-testing (HIVST) in the context of the COVID-19 crisis.

The study was conducted across eight socio-economically and geographically diverse sites in Manicaland province, selected to reflect the province’s main residential strata. These included a tea estate (Eastern Highlands), inhabited by agricultural laborers; rural: Bonda, a remote rural village with subsistence farming; forestry area, Selbourne with forestry-based livelihoods; towns: Nyazura and Nyanga, small towns with mixed economies; roadside settlement: Watsomba, a transient community along major transport routes; urban, Sakubva and Hobhouse, urban areas within the city of Mutare. The study area HIV prevalence is estimated at 11.3% in the 2018–2019 survey [17].

### Recruitment of study participants

Participants were recruited using stratified random sampling based on household enumeration within the selected sites. Updated census listings maintained by the Manicaland Centre for Public Health Research were used to systematically sample households. All individuals aged 15 years and older residing in the selected households were eligible to participate. Data were used from three rounds of the survey with 9,542 participants from 5,705 households from July 2018 to December 2019, 8,143 participants from 3,918 households from February to July 2021, and 9,240 participants from 4,709 households in July 2022 to January 2023. The reduction in households from 5,705 (2018–2019) to 3,918 (2021) and 4,709 (2022–2023) resulted from temporary fieldwork suspensions during COVID-19 restrictions, increased migration from rural to urban areas, and a shift to phone-based follow-up of reachable participants only.

In cases where eligible individuals were not present at the time of the household visit, fieldworkers made at least two return visits before recording non-response [39].

### Data collection

Data were collected using structured questionnaires. In Round 7 (2018–2019), interviews were conducted face-to-face by trained fieldworkers. Round 8 (2021) shifted to telephone interviews due to COVID-19 restrictions, using a shortened questionnaire to reduce participant burden. Round 9 (2022–2023) used a hybrid approach (telephone and face-to-face) based on participant preference and availability.

Study questionnaires were developed through an iterative process informed by prior Manicaland survey and established HIV surveillance. Initial drafts were reviewed by the study team to ensure alignment with the study objectives and relevance to local HIV service delivery contexts. Questionnaires were piloted in non-study communities to assess clarity, cultural appropriateness, and respondent burden. Feedback from pilot testing was used to refine wording, response options, and skip patterns. Questionnaires were developed in English, translated into Shona, and independently back-translated for accuracy. To maintain comparability across survey rounds, questionnaire items were standardized over time, with only minor modifications introduced to reflect changes in HIV service delivery modalities. The full questionnaires are publicly available through the Manicaland Centre for Public Health Research [39]. The data collected included socio-demographic characteristics, HIV testing history, HIV self-testing (HIVST) awareness and use, perceived testing barriers, and PrEP awareness and use. Fieldworkers were trained in ethics and confidentiality, with quality assurance through supervision, double entry, and electronic checks. In the 2018–2019 survey, younger individuals were oversampled to ensure adequate representation of youth, males aged 15–29 years and females aged 15–24 years were included from all households, whereas in other rounds, eligibility household members were randomly selected from two-thirds of households.

Perceived barriers to HIV testing were measured using self-reported measures capturing individual perceptions of structural, social, and service-related constraints to accessing HIV testing services. Participants were asked whether specific factors had ever prevented or discouraged them from testing for HIV. Barrier items were analyzed as binary variables (yes/no). “Lack of privacy” referred to concerns that HIV testing could not be conducted confidentially, including fears of being seen at the testing facility. “Judgmental staff” captured perceptions of negative or stigmatizing attitudes from healthcare providers, such as feeling blamed, disrespected, or treated harshly because of perceived risk behaviors. The barrier “not appropriate to go there” reflected perceived social or normative constraints on attending HIV testing services, including beliefs that HIV testing sites were unsuitable for certain groups or that testing would imply immoral or inappropriate behavior.

HIV self-testing knowledge and awareness were defined as having ever heard of HIV self-testing. This was assessed using a binary survey item asking participants whether they had ever heard of HIVST prior to the interview (yes/no). The HIVST use was defined as ever having used an HIV self-test. This was measured using a binary survey item asking participants whether they had ever used an HIV self-test at any point prior to the interview (yes/no). No restriction to use within the last 12 months was applied.

### Statistical analysis

Data were analyzed using R [40] and the tidyverse package [41,42]. Descriptive statistics summarized sociodemographic characteristics, HIV testing status, HIVST knowledge and use, and perceived barriers to HIV testing across all three survey rounds (2018–2019, 2021, and 2022–2023). Given the oversampling in 2018–2019 round, all analyses were adjusted for age group, and regression models were estimated separately for each survey round to ensure that estimates of association were not biased by different age sampling. Analyses were stratified by study site, age group and sex to reduce potential sampling bias. Chi-square tests were used to assess associations between sociodemographic factors and key outcomes such as ever tested for HIV, knowledge of HIVST and ever used HIVST. Multivariable logistic regression analyses were conducted to examine the association between predictor variables and the likelihood of testing for HIV, knowledge of HIVST, and Usage of HIVST spanning pre-COVID-19 (2018–2019), during COVID-19 (2021), and post-COVID-19 (2022–2023) periods.

For individuals who participated in multiple survey rounds, panel analysis was used to assess within-individual changes in HIV testing behavior over time, accounting for correlation between repeated measures. Cross-sectional analyses were conducted on each survey round to estimate population-level associations. Generalized linear mixed-effects models (GLMMs) were used to account for clustering at the village level and repeated measures for individuals participating in multiple rounds. Random intercepts were included for village and individual IDs to address intra-cluster correlation and longitudinal dependencies. Fixed effects included sociodemographic variables, marital status, infection risks and study site location.

Variables for the multivariable model were selected based on significant associations observed in univariable analyses (p < 0.05). To control for multiple testing, Benjamini–Hochberg (BH) corrections were applied to adjust *p*-values. Model fit was evaluated using the Akaike Information Criterion (AIC), Bayesian Information Criterion (BIC), and Intraclass Correlation Coefficients (ICCs).

Missing data were handled using complete-case analysis in regression. Observations with missing values for variables included in a given model were excluded from analysis. No imputation was performed, as the proportion of missing data across key exposure and outcome variables was low and missingness did not exhibit systematic patterns across survey rounds or key sociodemographic characteristics. All analyses were based on available complete observations.

A sensitivity sub-analysis restricted to repeat participants (n = 3,043) was conducted to assess the robustness of the findings. Given the observational design and evolving cohort composition, results are interpreted as associations rather than causal effects.

## Results

### Study participants demographic characteristics

Significant shifts in demographic and behavioral characteristics were observed across the study periods. Urban residence participation increased from 16.4% to 24.0%, while rural residence representation decreased from 15.5% to 12.4% (*P* < 0.001). Female participation slightly declined from 58.3% to 56.5% (*P* = 0.037), and the proportion of participants aged >45 years rose from 27.7% to 28.9%, with a decrease among those aged 18–45 years from 58.1% to 57.2% (*P* < 0.001). Educational attainment improved, with tertiary education rising from 5.7% to 9.2% (*P* < 0.001), and unemployment decreased from 43.2% to 34.7% (*P* < 0.001). Cohabitation increased from 68.4% to 73.9% (*P* < 0.001). Table 1 presents the demographic and behavioral characteristics of the study population.

**Table 1:** Demographic and behavioral characteristics of the study population in Manicaland, Zimbabwe: Pre-COVID-19 (2018–2019), During COVID-19 (2021), and post-COVID-19 (2022–2023).

Study Period		Pre- COVID-19 (2018-2019)	During COVID-19 (2021)	Post- COVID-19 (2022-2023)	
Year		(N = 9542)	(N = 8143)	(N = 9240)	*P-*value^1^
Site	**Commercial Forestry plantation area**	1349 (14.1)	921 (11.3)	1113 (12)	<0.001
	**Roadside settlement**	1659 (17.4)	1404 (17.2)	1514 (16.4)	
	**Rural (village)**	1476 (15.5)	1129 (13.9)	1144 (12.4)	
	**Tea estate**	997 (10.4)	881 (10.8)	934 (10.1)	
	**Town**	2500 (26.2)	2305 (28.3)	2318 (25.1)	
	**Urban (Mutare)**	1561 (16.4)	1503 (18.5)	2217 (24)	
Sex	**Female**	5559 (58.3)	4625 (56.8)	5223 (56.5)	0.037
	**Male**	3983 (41.7)	3518 (43.2)	4017 (43.5)	
Age Group	**<18 years**	1355 (14.2)	1179 (14.5)	1287 (13.9)	<0.001
	**18-45 years**	5541 (58.1)	4808 (59)	5282 (57.2)	
	**>45 years**	2646 (27.7)	2156 (26.5)	2671 (28.9)	
Education	**Primary**	1931 (20.2)	2305 (28.3)	1751 (19)	<0.001
	**Secondary**	7071 (74.1)	5215 (64.0)	6643 (71.9)	
	**Tertiary**	540 (5.7)	623 (7.7)	846 (9.2)	
Occupation	**Subsistence Farming**	750 (7.9)	669 (8.2)	1323 (14.3)	<0.001
	**Small-scale business**	871 (9.1)	1083 (13.3)	1222 (13.2)	
	**Civil servant**	268 (2.8)	358 (4.4)	553 (6)	
	**Estate worker**	1017 (10.7)	749 (9.2)	699 (7.6)	
	**Construction Worker**	113 (1.2)	188 (2.3)	426 (4.6)	
	**Unemployed**	4124 (43.2)	3430 (42.1)	3208 (34.7)	
	**Student**	1659 (17.4)	1089 (13.4)	1235 (13.4)	
	**others**	740 (7.8)	577 (7.1)	574 (6.2)	
Marital status	**divorced**	294 (3.1)	256 (3.1)	335 (3.6)	<0.001
	**Not Married**	3019 (31.6)	2095 (25.7)	2410 (26.1)	
	**separated**	351 (3.7)	360 (4.4)	390 (4.2)	
	**still in union**	5270 (55.2)	4874 (59.9)	5381 (58.2)	
	**widowed**	608 (6.4)	558 (6.9)	724 (7.8)	
Cohabiting	**No**	3019 (31.6)	2095 (25.7)	2410 (26.1)	<0.001
	**Yes**	6523 (68.4)	6048 (74.3)	6830 (73.9)	

^1^The table presents the distribution of percentages for each characteristic stratified by survey year. *P* -values derived from Chi-square tests assessing differences in distributions across survey rounds.

### HIV testing patterns

**Fig 1:**
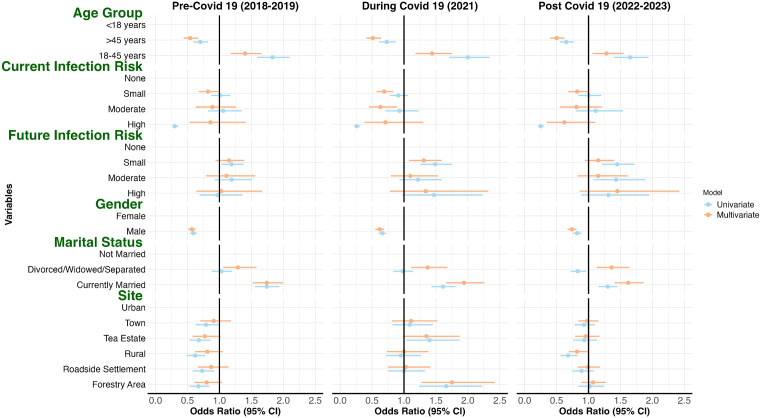
Association of HIV testing by sex, age group, study site, infection risk, and marital status pre (2018–2019), during (2021), and post-COVID-19 (2022–2023). Forest plot of odds ratios (ORs) and 95% confidence intervals (CIs) for the likelihood of HIV testing derived from a generalized linear mixed-effects model.

Men consistently had lower odds of reporting having tested for HIV compared with women. Adjusted analyses confirmed these disparities in all rounds. In 2018–2019, males had lower odds of reporting having tested for HIV (AOR = 0.57, 95% CI: 0.51–0.63, *P* < 0.001, Fig 1). During 2021, this disparity persisted (AOR = 0.62, 95% CI: 0.55–0.69; *P* < 0.001, Fig 1). By 2022–2023, the gap narrowed slightly but remained significant (AOR = 0.74, 95% CI: 0.67–0.81; *P* < 0.001, Fig 1). Fig 1 illustrates these findings with a forest plot of ORs for HIV testing by sex, age, site, and marital status from 2018-2023 (Fig 1).

Geographic and demographic factors further influenced testing likelihood. Rural residents had lower odds of HIV testing compared to urban residents, particularly post-COVID-19 (AOR = 0.82, 95% CI: 0.69–0.99, *P* = 0.034). Participants aged 18–45 years were more likely to test compared to those <18 years (post-COVID-19, AOR = 1.28, 95% CI: 1.06–1.55, *P* = 0.010), while those >45 years consistently had lower odds (post-COVID-19, AOR = 0.50, 95% CI: 0.40–0.62, *P* < 0.001). Being married was associated with higher testing odds across all periods (post-COVID-19, AOR = 1.62, 95% CI: 1.41–1.86, *P* < 0.001) (**S1 Table****).**

Comparison of multivariable logistic regression models with and without a village-level random effect showed that ICCs were consistently low across survey rounds, indicating minimal clustering at the village level. Inclusion of the village effect did not improve model fit, as most of the variation in the outcome was attributable to individual-level factors rather than village-level differences. Pre-Covid-19, the model yielded an AIC of 9348.611 and a BIC of 9472.276. The ICC at the village level was 0.01, indicating that only 1% of the total variance in the outcome was attributable to clustering at the village level. During Covid-19, model fit indices were similar AIC = 8786.213, BIC = 8908.945, with an ICC of 0.018, suggesting a slightly higher degree of between-village variance. Post-Covid-19, the model produced an AIC of 10087.680 and a BIC of 10212.450, with an ICC of 0.0001, indicating minimal clustering effects at the village level.

### Factors associated with HIV testing in repeat participants

Follow-up sensitivity sub-analysis analysis restricted to repeat participants (n = 3043) showed that the 18–45 year age group was more likely to report HIV testing in pre- COVID-19 (2018–2019, OR 1.60, 95% CI 1.01–2.53), but less likely in During COVID-19 (2021, OR: 0.69, 95% CI 0.50–0.96) and post- COVID-19 (2022–2023, OR:0.56, 95% CI 0.38–0.81). Men were associated with lower testing pre-COVID-19 (OR 0.67–0.70, all *p* < 0.001), and, became non-significant in 2021 (p = 0.393), and regained significance post-COVID (2022–2023) 2022–2023 (OR 0.80, *p* = 0.013) compared to women. Study site, marital status, and perceived HIV risk showed consistent associations across survey rounds (**S2 Table****).**

### Perceived barriers to HIV testing

The percentage of men perceiving barriers to HIV testing peaked during the COVID-19 pandemic (2021) and declined by 2022–2023, except for “not appropriate to go there,” which remained elevated at 7.1% (*P* < 0.0001). “Judgmental staff” increased from 11.1% in 2018–2019 to 13.2% in 2021 (*P* < 0.0001) before declining to 11.2% in 2022–2023. “High costs,” the least reported barrier, rose from 2.6% to 7.4% in 2021 but dropped to 3.6% by 2022–2023 (*P* < 0.0001). “Distance/travel difficulties” decreased from 16.0% to 10.1% over the study period (*P* < 0.0001).

Overall, men were substantially more likely than women to report “judgmental staff “(24.5% vs 4.7%) and “lack of privacy:” (21.5% vs 5.1%) as barriers to HIV testing, representing roughly a fourfold difference.

**Fig 2:**
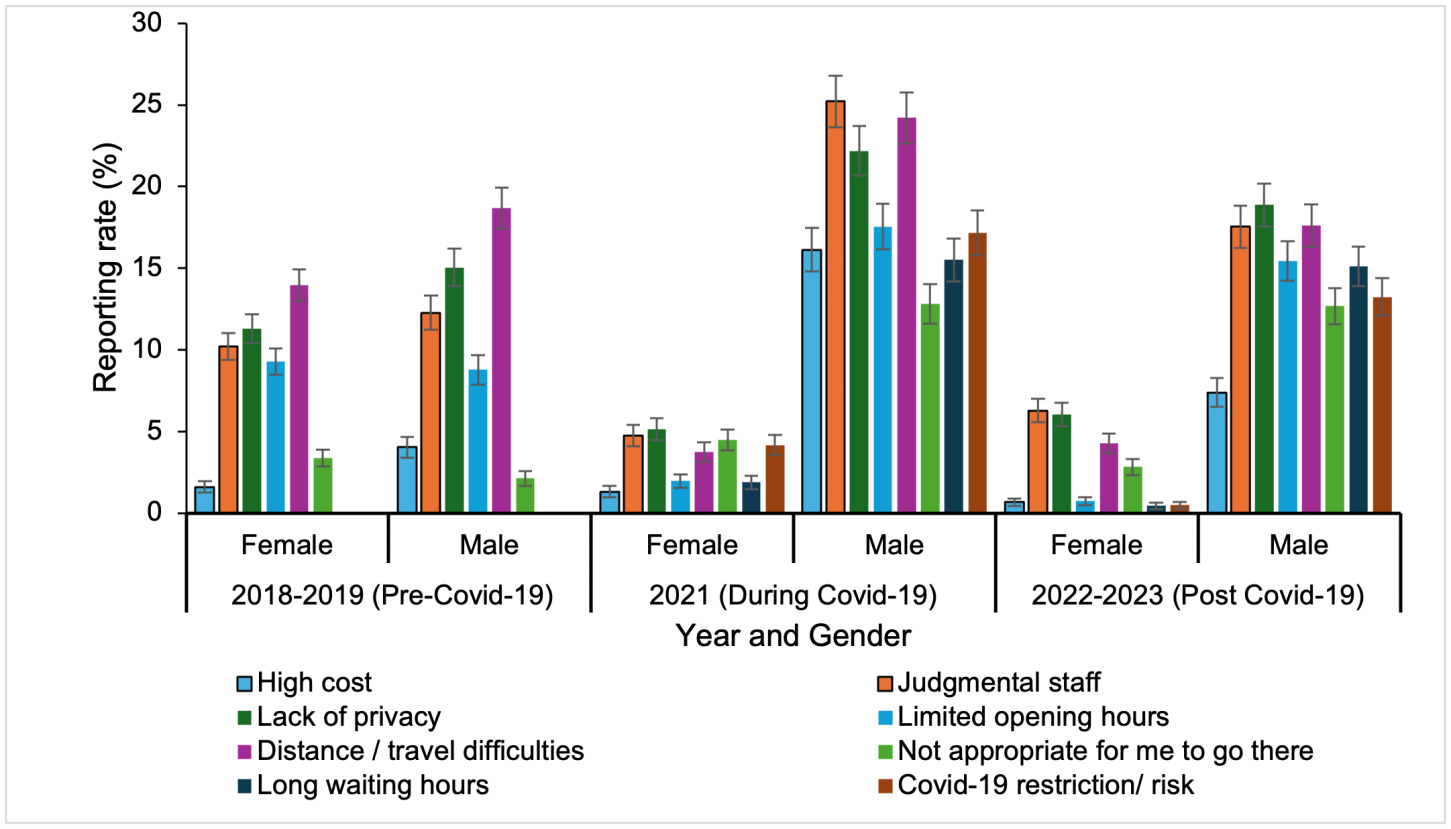
Perceived barriers to HIV testing by age group across the years from 2018-2023. Error bars represent 95% CIs.

Distance and travel challenges remained persistent barriers, particularly among older adults. Comparing the pre-COVID (2018–2019) and COVID (2021) survey rounds, the proportion reporting “distance/travel difficulties” increased among men from 18.7% to 23.5%, while it decreased among women from 14.0% to 3.7% (P = 0.002). Over the same period, reports of “lack of privacy/confidentiality” increased among men (15.0% to 21.5%) but declined among women (11.3% to 5.1%; *P* = 0.032). In the post-COVID period (2022–2023), these gender disparities persisted, with men continuing to report higher barriers than women (e.g., distance/travel difficulties: 17.6% vs. 4.3%, *P* = 0.002) (Fig 2).

Older adults (>45 years) consistently reported higher barriers, particularly “distance/travel difficulties” (20.6% in 2018–2019 vs. 14.8% for 18–45 years, *P* < 0.0001). While reports of this barrier decreased during the COVID-19 pandemic for all groups (e.g., 11.9% for both 18–45 years old and >45 years old years in 2021), the trend reversed slightly in the post-COVID period for those <18 years old (14.0%) and >45 (9.2%) (*P* < 0.0001). “High cost” was consistently the least reported perceived barrier. In 2018–2019, only 2.1% of <18-year-olds reported this issue, compared to 3.0% of 18–45-year-olds and 2.1% of >45-year-old. During the pandemic, the percentage increased to 7.9% for <18-year-olds and 7.3% for 18–45-year-old but remained comparatively low for >45 years old at 7.5%. In the post-COVID period, reporting percentage decreased across all age groups (*P* < 0.0001) (Table 2**).**

**Table 2:** Perceived barriers to HIV testing by sex and age group in Manicaland, Zimbabwe: Pre-COVID-19 (2018–2019), During COVID-19 (2021), and post-COVID-19 (2022–2023).

Barriers			2018-2019 (Pre-Covid 19)		2021 (During Covid-19)		2022-2023 (Post-Covid-19)		*P*-value^1^
			Enrolment (N)	Reporting n(n/N)	Enrolment (N)	Reporting n(n/N)	Enrolment (N)	Reporting n(n/N)	
Overall	**High costs**		**8722**	230 (2.6)	6977	518 (7.4)	7748	278 (3.6)	<0.0001
	**Judgmental staff**			967 (11.1)		918 (13.2)		866 (11.2)	<0.0001
	**Lack of privacy /confidentiality**			1125 (12.9)		846 (12.1)		900 (11.6)	0.041
	**Limited opening hours**			791 (9.1)		585 (8.4)		552 (7.1)	<0.0001
	**Travel difficulties**			1394 (16.0)		849 (12.2)		781 (10.1)	<0.0001
	**Not appropriate to go there**			247 (2.8)		549 (7.9)		551 (7.1)	<0.0001
	**Long waiting hours**					523 (7.5)		528 (6.8)	<0.0001
	**COVID-19 restriction/ risk**					662 (9.5)		467 (6.0)	<0.0001
Sex	**High costs**	**Male**	3724	150 (4.0)	2980	466 (15.6)	3370	279 (7.4)	0.008
		**Female**	4998	80 (1.6)	3997	52 (1.3)	4378	29 (0.7)	
	**Judgmental staff**	**Male**	3724	457 (12.3)	2980	729 (24.5)	3370	591 (17.5)	0.005
		**Female**	4998	510 (10.2)	3997	189 (4.7)	4378	275 (6.3)	
	**Lack of privacy /confidentiality**	**Male**	3724	560 (15.0)	2980	641 (21.5)	3370	636 (18.9)	0.032
		**Female**	4998	565 (11.3)	3997	205 (5.1)	4378	264 (6.0)	
	**Limited opening hours**	**Male**	3724	327 (8.8)	2980	507 (17.0)	3370	520 (15.4)	0.802
		**Female**	4998	464 (9.3)	3997	78 (2.0)	4378	32 (0.7)	
	**Distance / travel difficulties**	**Male**	3724	696 (18.7)	2980	700 (23.5)	3370	594 (17.6)	0.002
		**Female**	4998	698 (14.0)	3997	149 (3.7)	4378	187 (4.3)	
	**Not appropriate to go there**	**Male**	3724	79 (2.1)	2980	370 (12.4)	3370	427 (12.7)	0.429
		**Female**	4998	168 (3.4)	3997	179 (4.5)	4378	124 (2.8)	
	**Long waiting hours**	**Male**			2980	448 (15.0)	3370	509 (15.1)	<0.0001
		**Female**			3997	75 (1.9)	4378	19 (0.4)	
	**COVID-19 restriction/ risk**	**Male**			2980	496 (16.6)	3370	446 (13.02)	<0.0001
		**Female**			3997	166 (4.2)	4378	21 (0.5)	
Age group	**High costs**	**<18**	1779	37 (2.1)	1179	93 (7.9)	1286	76 (5.9)	<0.0001
		**18-45**	5286	158 (3.0)	4805	351 (7.3)	1180	46 (3.9)	
		**>45**	1657	35 (2.1)	993	74 (7.5)	5282	156 (3.0)	
	**Judgmental staff**	**<18**	1779	191 (10.7)	1179	162 (13.7)	1286	178 (13.8)	<0.0001
		**18-45**	5286	588 (11.1)	4805	624 (13.0)	1180	123 (10.4)	
		**>45**	1657	188 (11.3)	993	132 (13.3)	5282	565 (10.7)	
	**Lack of privacy /confidentiality**	**<18**	1779	201 (11.3)	1179	141 (12.0)	1286	173 (13.5)	<0.0001
		**18-45**	5286	720 (13.6)	4805	595 (12.4)	1180	123 (10.4)	
		**>45**	1657	204 (12.3)	993	110 (11.1)	5282	604 (11.4)	
	**Limited opening hours**	**<18**	1779	140 (7.9)	1179	101 (8.6)	1286	124 (9.6)	<0.0001
		**18-45**	5286	485 (9.2)	4805	389 (8.1)	1180	89 (7.5)	
		**>45**	1657	166 (10.0)	993	95 (9.6)	5282	339 (6.4)	
	**Distance / travel difficulties**	**<18**	1779	269 (15.1)	1179	157 (13.3)	1286	180 (14.0)	<0.0001
		**18-45**	5286	783 (14.8)	4805	574 (11.9)	1180	117 (9.9)	
		**>45**	1657	342 (20.6)	993	118 (11.9)	5282	484 (9.2)	
	**Not appropriate to go there**	**<18**	1779	66 (3.7)	1179	128 (10.9)	1286	171 (13.3)	<0.0001
		**18-45**	5286	121 (2.3)	4805	342 (7.1)	1180	90 (7.6)	
		**>45**	1657	60 (3.6)	993	79 (8.0)	5282	290 (5.5)	
	**Long waiting hours**	**<18**			1179	90 (7.6)	1286	125 (9.7)	<0.0001
		**18-45**			4805	357 (7.4)	1180	92 (7.8)	
		**>45**			993	76 (7.7)	5282	311 (5.9)	
	**COVID-19 restriction/ risk**	**<18**			1179	117 (9.9)	1286	112 (8.7)	<0.0001
		**18-45**			4805	450 (9.4)	1180	77 (6.5)	
		**>45**			993	95 (9.6)	5282	278 (5.3)	

* *P* -values from Chi-square tests.

### Barriers to HIV testing by testing status and survey year

A follow-up analysis examining trends in reported perceived barriers among those not testing for HIV indicated that most barriers did not differ significantly by testing status or showed significant differences only in specific survey years (Table 3). In contrast, the perception that testing sites were “not appropriate to go there” differed consistently and strongly by testing status across all survey years (*p* < 0.001), with substantially higher reporting among those who had never tested for HIV.

**Table 3:** Perceived barriers to HIV testing by HIV testing status (ever tested vs. never tested) across survey period Pre-COVID-19 (2018–2019), During COVID-19 (2021), and post-COVID-19 (2022–2023).

	Pre-Covid 19 (2018–2019)			During Covid 19 (2021)			Post-Covi-19 (2022–2023)		
Barrier	Not tested (N = 1,719)	Tested (N = 6,990)	p-value	Not tested (N = 607)	Tested (N = 6,364)	p-value	Not tested (N = 921)	Tested (N = 6,827)	p-value
High costs	52 (3.0)	178 (2.5)	0.306	30 (4.9)	488 (7.7)	0.018	34 (3.7)	244 (3.6)	0.931
Judgmental staff	167 (9.7)	797 (11.4)	0.055	72 (11.9)	845 (13.3)	0.355	95 (10.3)	771 (11.3)	0.407
Lack of privacy/confidentiality	188 (10.9)	933 (13.3)	0.008	55 (9.1)	789 (12.4)	0.019	95 (10.3)	805 (11.8)	0.21
Limited opening hours	115 (6.7)	673 (9.6)	0.0002	35 (5.8)	549 (8.6)	0.018	60 (6.5)	492 (7.2)	0.485
Distance/travel difficulties	277 (16.1)	1,113 (15.9)	0.875	62 (10.2)	786 (12.4)	0.141	62 (6.7)	466 (6.8)	0.971
Not appropriate to go there	124 (7.2)	122 (1.7)	<0.0001	82 (13.5)	467 (7.3)	<0.0001	125 (13.6)	426 (6.2)	<0.0001
Long waiting hours†	—	—		30 (4.9)	492 (7.7)	0.015	62 (6.7)	466 (6.8)	0.971
COVID-19 restriction/risk†	—	—		46 (7.6)	615 (9.7)	0.011	53 (5.8)	414 (6.1)	0.766

### HIVST awareness and use

HIVST awareness increased significantly between 2018 and 2023, particularly among men (38.4% to 59.4%) (vs. 19.1% to 48.4% for females, *P* < 0.001). Despite this growing awareness, HIVST use remained low, increasing only modestly from 2.1% to 5.9% among men and from 3.9% to 12.2% among women (*P* < 0.001).

Urban areas (59.9%) and forestry areas (65.3%) reported the largest awareness by 2022–2023 compared to rural areas (29.3%). The 18–45 years age group had the highest awareness by 2023 (60.0%, *P* < 0.001) (**S3 Table**).

The GLMMs identified males (AOR = 2.21, 95% CI: 2.09–2.33, *P*< 0.0001) and those aged 18–45 years (AOR = 2.34, 95% CI: 2.17–2.53, *P* < 0.0001) as more likely to know about HIVST compared to those under 18 years, while rural residents had lower odds (AOR = 0.41, 95% CI: 0.32–0.53, *P* < 0.0001) (**S4 Table**)

The percentage of respondents reporting usage of HIVST increased significantly between 2.9% Pre- COVID-19 to 9.3% post-COVID-19 though it remained much lower than the percentage reporting they were aware of HIVST (9.3% vs 53.1% post-COVID-19 period) The 18–45 years age group led in usage (10.7% by 2023, *P*< 0.001) (**S3 Table**). The GLMMs showed females (AOR = 1.92, 95% CI: 1.67–2.22, *P* < 0.001), the 18–45 years age group (AOR = 2.76, 95% CI: 2.00–3.83, *P* < 0.001), and the post-COVID-19 period (AOR = 3.31, 95% CI: 2.57–4.25, *P* < 0.0001) as predictors of higher HIVST usage, with rural residents showing lower odds (AOR = 0.59, 95% CI: 0.37–0.95, *P* = 0.029) (**S5 Table****).**

## Discussion

This study demonstrates persistent sex disparities in HIV testing in Zimbabwe, with men reporting higher perceived barriers and lower testing rates. These differences intensified during the COVID-19 pandemic, when limited-service availability, stigma, and privacy concerns were heightened.

Although awareness of HIVST increased during this period, particularly in urban settings, actual uptake remained low among men, indicating that awareness alone is insufficient to overcome access barriers. Findings revealed significantly lower odds of HIV testing among men in 2022–2023 (AOR = 0.74, 95% CI: 0.67–0.81), with the pandemic exacerbating longstanding service limitations, including lack of privacy and restricted availability. Our findings align with existing literature from sub-Saharan Africa, highlighting sex disparities in HIV testing. Men’s lower testing rates reflect social norms, stigma, and limited healthcare engagement, while women’s higher uptake is likely driven by routine access to healthcare through maternal and child health services [8,10,14,43]. These findings highlight the importance of targeted efforts to close sex gaps in HIV testing and to build more resilient health systems. They also provide valuable insights for ongoing initiatives like the IMPERATIVE trial (NCT06370923) a large, ongoing cluster-randomized study leveraging male peer networks to enhance HIVST and PrEP uptake in Zimbabwe, providing an operational framework aligned with the current study’s findings.

The COVID-19 pandemic significantly disrupted testing, with barriers peaking in 2021, especially among men, who reported concerns like “judgmental staff” (13.2%). These align with regional reports of service interruptions due to lockdowns and staff shortages [44,45]. Urban residents faced heightened barriers and lower odds of testing during the pandemic in 2021, possibly due to stricter health restrictions, increased stigma, or anxiety about visiting facilities during the pandemic [46,47]. These disruptions, driven by lockdowns and staffing shortages disproportionately affected urban residents, who faced stricter restrictions and heightened stigma [44–47],. Although most barriers declined by 2022–2023, perceptions of stigma remained, particularly among men (12.7% vs. 2.8% for women) reporting testing as “not appropriate.” Testing rebounded in urban areas possibly due to younger, more mobile populations [48], but rural areas and tea estates showed slower recovery, highlighting uneven health system resilience. The longitudinal analysis of repeat participants shows a shift in HIV testing behavior. Among the aged 18–45-year age group, the reversal of association from higher likelihood of testing pre-pandemic to lower likelihood during and post-pandemic suggests that younger adults faced greater barriers to testing during COVID-19, including mobility restrictions, clinic closures, and economic or social challenges. Men in 2021 did not show statistical significance in testing during the Covid-19, indicate that pandemic-era interventions may have partially mitigated gender disparities. However, post-pandemic data show that the male disadvantage in testing persists, highlighting the continued importance of male-focused outreach.

Age-specific barriers and testing patterns from the current study show that younger individuals (<18 years) exhibited the lowest testing rates (36.9% to 45.2%) and highest perceived barriers, particularly “not appropriate to go there” (13.3% in 2022–2023). These findings are consistent with HIV testing studies in Zambia (53%) and Nigeria (23%) where stigma, lack of youth-friendly services, and parental consent requirements hinder testing [49,50]. This trend is concerning, as regular testing is critical for monitoring treatment efficacy and preventing transmission. These findings highlight the need for education campaigns emphasizing the importance of testing, and targeted outreach to re-engage older populations. In addition, findings of “not appropriate to go there” differing consistently and strongly between respondents who had ever tested and those who had never tested for HIV, with higher reporting among non-testers, indicate that while many barriers reflect system-wide service challenges, certain social acceptability or stigma-related barriers may act as key deterrents to HIV testing.

HIVST awareness increased, particularly among males (38.4% to 66.7%), reflecting the success of awareness campaigns and community-based distribution efforts [51]. However, usage remained low, with females (2.7% to 9.8%) outpacing males (2.1% to 5.9%), and rural areas lagging the urban areas. These trends align with studies identifying stigma, cost, and lack of linkage to care as barriers to HIVST uptake [30,31]. The 18–45 years age group showed the highest usage (10.7%), while youth (<18 years) improved from 0% to 6.1%, indicating HIVST’s potential to reach younger populations, consistent with findings from Malawi and Zimbabwe [52,53]. Higher HIVST usage in tea estates (9.0%) compared to roadside settlements (5.2%) supports the need for targeted kit distribution in remote settings [26,27]. Peer-led HIVST programs, which leverage social networks to increase uptake, offer a promising solution, as demonstrated in prior studies [28,29]. The findings of the current study highlight the need for context-specific interventions, such as mobile testing units, community health worker outreach, or peer-led HIVST programs, to enhance access in remote settings [28]. The current study shows that low uptake of HIVST despite increased awareness, necessitates integration of counseling, linkage-to-care mechanisms, and sex-sensitive peer-led models to enhance acceptability and usage. Such models, such as the IMPERATIVE trial being tested in the same study setting, align with WHO recommendations for community-based HIVST scale-up and peer-led distribution models [54].

The longitudinal nature of the data allows assessment of temporal changes in the HIV testing uptake patterns. Following the same individuals across multiple survey rounds enables detection of within-person changes over time, improving causal inference compared to repeated cross-sectional analyses. While sex inequalities in testing are well established, our findings reveal how these barriers evolved before, during, and after COVID-19, providing important insights for post-pandemic recovery planning. The inclusion of diverse geographic settings (urban, rural, tea estates, and forestry areas) enhances the applicability of findings within Zimbabwe’s varied contexts. However, limitations must be acknowledged. The shift to telephone interviews in 2021 may have altered selection bias, as participants with phone access may differ socioeconomically from those without. Additionally, the study did not assess linkage to care post-HIVST, limiting insights into treatment outcomes. Furthermore, in the longitudinal panel analysis, only individuals participating in all three rounds were included, which may be affected by selection bias, as the panel excludes individuals lost to follow-up and could influence self-reporting of HIV testing. Temporal changes may also reflect unmeasured external factors, such as local COVID-19 restrictions or outreach campaigns. To mitigate some of these issues, we employed rigorous statistical adjustments and stratified sampling to ensure representativeness. Adjustments for underlying demographic composition, survey mode, and repeat-tester robustness checks further strengthen confidence in observed patterns.

These findings have important implications for HIV prevention and public health. Longitudinal findings among repeat participants highlight that pandemic-related disruptions had differential effects by age and gender, and tailored interventions are needed to support younger adults and men in maintaining HIV testing uptake, particularly during public health emergencies. Persistent sex disparities necessitate male-focused interventions, such as peer-led HIVST programs and private, stigma-free testing options. Peer-led initiatives like the IMPERATIVE trial (NCT06370923) and the E-PREP trials (NCT03213366) [55] could offer scalable models to enhance testing and PrEP uptake among men. COVID-19 disruptions highlight the need for resilient health systems, including mobile testing, telehealth, and flexible service delivery [56]. Our data suggest that rural and forestry areas may require infrastructure improvements, including community health worker outreach and subsidized HIVST kits. Expanding HIVST access and strengthening linkage-to-care are critical to closing the awareness-usage gap and ensuring equitable and sustainable progress in HIV prevention across Zimbabwe.

## Conclusion

Men continue to report substantially higher barriers to HIV testing in Manicaland, Zimbabwe, associated with privacy, stigma, and healthcare providers. These disparities intensified during the COVID-19 pandemic, remain largely unaddressed, and continue to hinder the effectiveness of HIV prevention efforts. Although awareness of HIVST increased, uptake remains insufficient, highlighting the need for context-tailored interventions integrating counseling and community-based distribution. These challenges will be further compounded by declining global funding for HIV programs. Ensuring equitable access to HIV testing requires sustained investment in sensitive strategies, enhanced linkage-to-care systems, and adaptive strategies to buffer against future disruptions such as pandemic or funding shortfalls. Without urgent and focused investments, funding cuts combined with lingering impacts of the pandemic will continue to erode service availability and deepen the already existing disparities. This threatens to reverse progress made in HIV testing coverage and undermines the potential impact of interventions such as the IMPERATIVE trial. Addressing these disparities is a critical requirement for epidemic control in high-burden settings.

## Supporting information

S1 Table Factors Associated with Lifetime HIV Testing in Manicaland, Zimbabwe: Pre-COVID-19 (2018–2019), During COVID-19 (2021), and post-COVID-19 (2022–2023). The table presents the univariate and multivariate odds ratios (OR) with 95% confidence intervals (CI), False discovery rate (FDR) for various factors associated with ever testing for HIV, based on data from the 2018–2019, 2021, and 2022–2023 surveys. Variables include study site, sex, age group, marital status, perceived risk of infection, and future likelihood of infection. The results are presented for both univariate and multivariate models.
(DOCX)

S2 Table Factors associated with ever testing for HIV among participants followed across all three survey rounds (2018–2019, 2021, 2022–2023, n = 3,043). The table presents trends in knowledge and use of HIV self-testing (HIVST) among participants from 2019, 2021, and 2023, disaggregated by study site, sex, and age group. The table shows the number and percentage of individuals who reported having used HIVST, heard of HIVST but never used it, or never heard of HIVST in each year. *P*-values reflect the results of Chi-square tests and indicate whether the differences in knowledge and use across survey years within each subgroup are statistically significant.
(DOCX)

S3 Table Knowledge and Use of HIV Self-Testing (HIVST) Across the Years by Study Site, Gender, and Age Group: The table presents trends in knowledge and use of HIV self-testing (HIVST) among participants from 2018-2019, 2021, and 2022–2023, disaggregated by study site, gender, and age group. The table shows the number and percentage of individuals who had knowledge of HIVST and used HIVST.
(DOCX)

S4 Table Factors Associated with Knowledge of HIV Self-Testing: Results from Generalized Linear Mixed-Effects Models. Summary of univariate and multivariate regression analyses from generalized linear mixed-effects models evaluating factors associated with knowledge of HIV self-testing (HIVST). Odds ratios (ORs) and 95% confidence intervals (CIs) are reported for each variable, with p-values indicating the statistical significance of observed associations. The dependent variable was “Knowledge of HIVST.” Results are presented across study sites, sex, age groups, and survey rounds, with the reference categories for each variable noted. Multivariate models adjusted for potential confounders.
(DOCX)

S5 Table Factors Associated with usage of HIV Self-Testing: Results from Generalized Linear Mixed-Effects Models. Summary of univariate and multivariate regression analyses from generalized linear mixed-effects models evaluating factors associated with usage of HIV self-testing (HIVST). Odds ratios (ORs) and 95% confidence intervals (CIs) are reported for each variable, with p-values indicating the statistical significance of observed associations. The dependent variable was “Usage of HIVST.” Results are presented across study sites, sex, age groups, and survey rounds, with the reference categories for each variable noted. Multivariate models adjusted for potential confounders.
(DOCX)
