# Supplementary material for: Do men face greater barriers to accessing HIV testing services than women? Might HIV self-testing be the answer? Evidence from a longitudinal survey in east Zimbabwe (2018–2023)
Source: PLOS Glob Public Health. 2026 Mar 24;6(3):e0006125. doi: 10.1371/journal.pgph.0006125 (PMC13012501; doi:10.1371/journal.pgph.0006125)
Supplement: S1 Table — The table presents the univariate and multivariate odds ratios (OR) with 95% confidence intervals (CI), False discovery rate (FDR) for various factors associated with ever testing for HIV, based on data from the 2018–2019, 2021, and 2022–2023 surveys. Variables include study site, sex, age group, marital status, perceived risk of infection, and future likelihood of infection. The results are presented for both univariate and multivariate models. (DOCX) [file pgph.0006125.s001.docx]

**S1 Table: Factors Associated with HIV Testing in Manicaland, Zimbabwe: Pre-COVID-19 (2018–2019), During COVID 19 (2021), and post-COVID-19 (2022–2023)**

|  | **2018-2019 (Pre-Covid 19)** | | | | | | | **During Covid-19 (2021)** | | | | | | | | **Post-Covid19 (2022-2023** | | | | | | | |
| --- | --- | --- | --- | --- | --- | --- | --- | --- | --- | --- | --- | --- | --- | --- | --- | --- | --- | --- | --- | --- | --- | --- | --- |
| **Variable** | **Tested** | **Univariate** | **p-value** | **Multivariate** | | **p-value** | **FDR adjusted P-value** | **Tested** | **Univariate OR (95% CI)** | **p-value** | **Multivariate OR (95% CI)** | | **p-value** | **FDR adjusted p-value** | **Tested** | | **Univariate** | **p-value** | **Multivariate** | | ***P*-value** | **FDR adjusted p-value** |  |
|  | n (%) | **OR (95% CI)** |  | **SE** | **OR (95% CI)** |  |  | n (%) |  |  | SE | **OR (95% CI)** |  |  | n (%) | | **OR (95% CI)** |  | SE | **OR (95% CI)** |  |  |  |
| **Study site** |  |  |  |  |  |  |  |  |  |  |  |  |  |  |  | |  |  |  |  |  |  |  |
| **Roadside settlement** | 564 (43.8) | 0.73 (0.58–0.92) | 0.008 | 0.14 | 0.87 (0.66–1.14) | 0.307 | 0.145 | 556 (41.5) | 1.00 (0.75–1.33) | 0.99 | 0.16 | 1.03 (0.75–1.41) | 0.856 | 0.91 | 650 (48.1) | | 0.89 (0.74–1.08) | 0.227 | 0.09 | 0.99 (0.83–1.18) | 0.901 | 0.901 |  |
| **Rural** | 490 (39.4) | 0.62 (0.49–0.78) | <0.0001 | 0.14 | 0.81 (0.62–1.05) | 0.116 | 0.198 | 436 (41.0) | 0.95 (0.72–1.27) | 0.752 | 0.16 | 1.00 (0.73–1.38) | 0.996 | 0.996 | 443 (41.5) | | 0.68 (0.56–0.83) | <0.001 | 0.09 | 0.82 (0.69–0.99) | 0.034 | 0.095 |  |
| **Tea estate** | 387 (42.2) | 0.68 (0.53–0.86) | 0.0018 | 0.14 | 0.77 (0.58–1.02) | 0.07 | 0.149 | 426 (50.4) | 1.40 (1.04–1.87) | 0.025 | 0.17 | 1.35 (0.98–1.87) | 0.07 | 0.108 | 417 (49.2) | | 0.93 (0.76–1.13) | 0.454 | 0.1 | 0.96 (0.79–1.17) | 0.687 | 0.778 |  |
| **Town** | 906 (44.3) | 0.79 (0.63–0.99) | 0.041 | 0.13 | 0.91 (0.70–1.18) | 0.483 | 0.593 | 966 (44.5) | 1.09 (0.82–1.45) | 0.537 | 0.16 | 1.11 (0.81–1.52) | 0.503 | 0.61 | 1055 (49.4) | | 0.93 (0.78–1.10) | 0.386 | 0.08 | 0.98 (0.84–1.15) | 0.816 | 0.866 |  |
| **Forestry area** | 472 (40.1) | 0.67 (0.53–0.84) | <0.001 | 0.13 | 0.80 (0.61–1.04) | 0.092 | 0.175 | 456 (51.3) | 1.66 (1.24–2.22) | 0.0007 | 0.17 | 1.75 (1.27–2.42) | 0.001 | 0.001 | 559 (50.8) | | 1.02 (0.84–1.24) | 0.857 | 0.09 | 1.07 (0.89–1.27) | 0.454 | 0.574 |  |
| **Urban (Ref)** | 568 (50.8) | 1 | — |  | 1 | — |  | 517 (41.6) | 1 | — |  | 1 | — |  | 936 (50.8) | | 1 | — |  | 1 | — |  |  |
| **Gender** |  |  |  |  |  |  |  |  |  |  |  |  |  |  |  | |  |  |  |  |  |  |  |
| **Male** | 1112 (35.9) | 0.59 (0.54–0.65) | <0.0001 | 0.05 | 0.57 (0.51–0.63) | <0.0001 | <0.0001 | 2135 (48.5) | 0.66 (0.60–0.72) | <0.0001 | 0.05 | 0.62 (0.55–0.69) | <0.0001 | <0.0001 | 1605 (45.7) | | 0.82 (0.75–0.89) | <0.0001 | 0.05 | 0.74 (0.67–0.81) | <0.0001 | <0.0001 |  |
| **Female (Ref)** | 2275 (48.5) | 1 | — |  | 1 | — |  | 1222 (38.8) | 1 | — |  | 1 | — |  | 2455 (50.8) | | 1 | — |  | 1 | — |  |  |
| **Age group** |  |  |  |  |  |  |  |  |  |  |  |  |  |  |  | |  |  |  |  |  |  |  |
| **18–45 years** | 2552 (50.2) | 1.83 (1.59–2.10) | <0.0001 | 0.09 | 1.40 (1.18–1.66) | <0.0001 | <0.0001 | 2425 (52.7) | 2.00 (1.71–2.34) | <0.0001 | 0.1 | 1.44 (1.18–1.75) | 0.0003 | 0.001 | 2867 (56.8) | | 1.65 (1.40–1.94) | <0.0001 | 0.1 | 1.28 (1.06–1.55) | 0.01 | 0.033 |  |
| **>45 years** | 467 (27.9) | 0.70 (0.59–0.82) | <0.0001 | 0.11 | 0.54 (0.44–0.67) | <0.0001 | <0.0001 | 641 (29.7) | 0.73 (0.61–0.87) | 0.0004 | 0.12 | 0.51 (0.41–0.64) | <0.0001 | <0.0001 | 885 (34.1) | | 0.65 (0.55–0.77) | <0.0001 | 0.11 | 0.50 (0.40–0.62) | <0.0001 | <0.0001 |  |
| **<18 years (Ref)** | 368 (35.7) | 1 | — |  | 1 | — |  | 291 (36.6) | 1 | — |  | 1 | — |  | 308 (44.3) | | 1 | — |  | 1 | — |  |  |
| **Marital Status** |  |  |  |  |  |  |  |  |  |  |  |  |  |  |  | |  |  |  |  |  |  |  |
| **Divorced/Widowed/Separated** | 405 (36.2) | 1.03 (0.88–1.20) | 0.714 | 0.1 | 1.29 (1.06–1.58) | 0.01 | 0.003 | 439 (36.6) | 0.98 (0.83–1.14) | 0.756 | 0.11 | 1.37 (1.11–1.68) | 0.0032 | 0.006 | 591 (40.9) | | 0.83 (0.72–0.96) | 0.0098 | 0.09 | 1.36 (1.13–1.64) | 0.0011 | 0.005 |  |
| **Currently Married** | 2300 (48.6) | 1.74 (1.56–1.94) | <0.0001 | 0.07 | 1.74 (1.52–2.00) | <0.0001 | <0.0001 | 2322 (48.9) | 1.61 (1.43–1.81) | <0.0001 | 0.08 | 1.94 (1.66–2.26) | <0.0001 | <0.0001 | 2734 (51.8) | | 1.30 (1.16–1.45) | <0.0001 | 0.07 | 1.62 (1.41–1.86) | <0.0001 | <0.0001 |  |
| **Not Married (Ref)** | 682 (35.2) | 1 | — |  | 1 | — |  | 596 (37.2) | 1 | — |  | 1 | — |  | 735 (45.5) | | 1 | — |  | 1 | — |  |  |
| **Current infection risk** |  |  |  |  |  |  |  |  |  |  |  |  |  |  |  | |  |  |  |  |  |  |  |
| **High** | 170 (20.9) | 0.30 (0.26–0.36) | <0.0001 | 0.25 | 0.86 (0.53–1.41) | 0.559 | 0.594 | 174 (20.1) | 0.26 (0.22–0.31) | <0.0001 | 0.31 | 0.71 (0.38–1.30) | 0.264 | 0.37 | 185 (21.6) | | 0.25 (0.21–0.30) | <0.0001 | 0.29 | 0.62 (0.35–1.10) | 0.101 | 0.214 |  |
| **Moderate** | 126 (47.9) | 1.06 (0.82–1.35) | 0.673 | 0.18 | 0.89 (0.63–1.26) | 0.499 | 0.594 | 100 (46.5) | 0.93 (0.71–1.23) | 0.616 | 0.17 | 0.63 (0.45–0.89) | 0.002 | 0.012 | 82 (55.4) | | 1.11 (0.80–1.54) | 0.537 | 0.2 | 0.81 (0.55–1.21) | 0.306 | 0.473 |  |
| **Low** | 386 (46.7) | 1.01 (0.87–1.17) | 0.89 | 0.1 | 0.82 (0.68–1.00) | 0.05 | 0.142 | 344 (45.2) | 0.91 (0.78–1.06) | 0.211 | 0.09 | 0.69 (0.57–0.83) | <0.0001 | 0.0004 | 292 (52,2) | | 1.00 (0.84–1.20) | 0.967 | 0.1 | 0.82 (0.68–1.00) | 0.053 | 0.128 |  |
| **None (Ref)** | 2705 (45.9) | 1 | — |  | 1 | — |  | 2739 (48.0) | 1 | — |  | 1 | — |  | 3501 (51.7) | | 1 | — |  | 1 | — |  |  |
| **Future infection risk** |  |  |  |  |  |  |  |  |  |  |  |  |  |  |  | |  |  |  |  |  |  |  |
| **High** | 65 (45.4) | 0.97 (0.69–1.36) | 0.869 | 0.25 | 1.03 (0.64–1.67) | 0.897 | 0.897 | 50 (54.3) | 1.47 (0.97–2.23) | 0.071 | 0.28 | 1.34 (0.78–2.32) | 0.292 | 0.382 | 60 (57.7) | | 1.31 (0.89–1.95) | 0.176 | 0.26 | 1.45 (0.86–2.42) | 0.161 | 0.295 |  |
| **Moderate** | 138 (49.6) | 1.11 (0.83–1.47) | 0.499 | 0.17 | 1.11 (0.79–1.56) | 0.537 | 0.593 | 121 (51.7) | 1.22 (0.93–1.59) | 0.143 | 0.17 | 1.10 (0.79–1.53) | 0.565 | 0.64 | 129 (60.0) | | 1.43 (1.08–1.89) | 0.012 | 0.17 | 1.15 (0.83–1.61) | 0.401 | 0.569 |  |
| **Low** | 406 (50.0) | 1.15 (0.94–1.40) | 0.169 | 0.1 | 1.15 (0.94–1.39) | 0.169 | 0.261 | 381 (55.5) | 1.49 (1.26–1.75) | <0.0001 | 0.1 | 1.31 (1.08–1.59) | 0.0056 | 0.011 | 344 (60.1) | | 1.45 (1.21–1.72) | <0.0001 | 0.1 | 1.15 (0.94–1.40) | 0.173 | 0.295 |  |
| **None (Ref)** | 2671 (46.7) | 1 | — |  | 1 | — |  | 2670 (46.5) | 1 | — |  | 1 | — |  | 3381 (50.7) | | 1 | — |  | 1 | — |  |  |

The table presents the univariate and multivariate Proposition tested, odds ratios (OR) with 95% confidence intervals (CI), Standard error (SE), False discovery rate (FDR) for various factors associated with ever testing for HIV, based on data from the 2018-2019, 2021, and 2022-2023 surveys. Variables include study site, gender, age group, marital status, perceived risk of infection, and future likelihood of infection. The results are presented for both univariate and multivariate models.
