# Supplementary material for: Do men face greater barriers to accessing HIV testing services than women? Might HIV self-testing be the answer? Evidence from a longitudinal survey in east Zimbabwe (2018–2023)
Source: PLOS Glob Public Health. 2026 Mar 24;6(3):e0006125. doi: 10.1371/journal.pgph.0006125 (PMC13012501; doi:10.1371/journal.pgph.0006125)
Supplement: S2 Table — The table presents trends in knowledge and use of HIV self-testing (HIVST) among participants from 2019, 2021, and 2023, disaggregated by study site, sex, and age group. The table shows the number and percentage of individuals who reported having used HIVST, heard of HIVST but never used it, or never heard of HIVST in each year. P-values reflect the results of Chi-square tests and indicate whether the differences in knowledge and use across survey years within each subgroup are statistically significant. (DOCX) [file pgph.0006125.s002.docx]

**S2 Table: Factors associated with ever testing for HIV among participants followed across all three survey rounds (2018–2019, 2021, 2022–2023, n = 3,043)**

|  | **2018-2019 (Pre-Covid 19)** | | | | **During Covid-19 (2021)** | | | | **Post-Covid19 (2022-2023** | | | |
| --- | --- | --- | --- | --- | --- | --- | --- | --- | --- | --- | --- | --- |
| **Variable** | **Univariate**  **OR (95% CI)** | **p-value** | **Multivariate**  **OR (95% CI)** | **p-value** | **Univariate OR (95% CI)** | **p-value** | **Multivariate OR (95% CI)** | **p-value** | **Univariate**  **OR (95% CI)** | **p-value** | **Multivariate**  **OR (95% CI)** | ***P*-value** |
| **Study site** |  |  |  |  |  |  |  |  |  |  |  |  |
| **Roadside settlement** | 0.84 (0.62–1.14) | 0.259 | 1.01 (0.70–1.46) | 0.945 | 1.03 (0.70–1.50) | 0.891 | 1.19 (0.79–1.80) | 0.407 | 1.05 (0.80–1.37) | 0.743 | 1.25 (0.93–1.68) | 0.140 |
| **Rural** | 0.61 (0.44–0.84) | 0.002 | 0.72 (0.49–1.04) | 0.077 | 1.56 (1.05–2.30) | 0.026 | 1.55 (1.01–2.37) | 0.044 | 1.21 (0.91–1.60) | 0.184 | 1.32 (0.97–1.80) | 0.073 |
| **Tea estate** | 0.70 (0.52–0.95) | 0.020 | 0.91 (0.64–1.29) | 0.591 | 0.92 (0.64–1.32) | 0.652 | 0.95 (0.64–1.40) | 0.786 | 0.77 (0.59–1.00) | 0.052 | 0.94 (0.70–1.25) | 0.661 |
| **Town** | 0.76 (0.54–1.06) | 0.101 | 0.81 (0.55–1.19) | 0.280 | 1.27 (0.85–1.88) | 0.241 | 1.21 (0.78–1.86) | 0.392 | 1.12 (0.82–1.52) | 0.478 | 1.28 (0.92–1.80) | 0.147 |
| **Forestry area** | 0.91 (0.68–1.21) | 0.503 | 0.98 (0.70–1.38) | 0.924 | 1.16 (0.81–1.66) | 0.411 | 1.16 (0.79–1.71) | 0.454 | 0.96 (0.75–1.23) | 0.728 | 1.04 (0.79–1.36) | 0.791 |
| **Urban (Ref)** |  |  |  |  |  |  |  |  |  |  |  |  |
| **Gender** |  |  |  |  |  |  |  |  |  |  |  |  |
| **Male** | 0.67 (0.57–0.80) | <0.001 | 0.67 (0.55–0.82) | <0.001 | 0.70 (0.60–0.83) | <0.001 | 0.66 (0.55–0.79) | 9.09e-06 | 0.93 (0.80–1.09) | 0.393 | 0.80 (0.67–0.95) | 0.013 |
| **Female (Ref)** |  |  |  |  |  |  |  |  |  |  |  |  |
| **Age group** |  |  |  |  |  |  |  |  |  |  |  |  |
| **18–45 years** | 2.01 (1.40–2.89) | <0.001 | 1.60 (1.01–2.53) | 0.045 | 0.85 (0.67–1.09) | 0.213 | 0.69 (0.50–0.96) | 0.027 | 0.58 (0.42–0.80) | 0.001 | 0.56 (0.38–0.81) | 0.002 |
| **>45 years** | 0.72 (0.50–1.06) | 0.094 | 0.63 (0.39–1.03) | 0.064 | 0.48 (0.41–0.57) | <0.001 | 0.56 (0.46–0.67) | 2.43e-09 | 0.68 (0.55–0.83) | <0.001 | 0.70 (0.56–0.87) | 0.001 |
| **<18 years (Ref)** |  |  |  |  |  |  |  |  |  |  |  |  |
| **Marital Status** |  |  |  |  |  |  |  |  |  |  |  |  |
| **Divorced/Widowed/Separated** | 0.84 (0.61–1.14) | 0.268 | 1.06 (0.71–1.60) | 0.766 | 0.77 (0.58–1.01) | 0.059 | 1.15 (0.79–1.66) | 0.476 | 0.54 (0.41–0.71) | <0.001 | 1.06 (0.75–1.49) | 0.742 |
| **Currently Married** | 1.47 (1.14–1.89) | 0.003 | 1.50 (1.07–2.09) | 0.018 | 1.41 (1.12–1.77) | 0.003 | 1.66 (1.23–2.25) | 0.001 | 0.91 (0.73–1.15) | 0.444 | 1.30 (0.99–1.71) | 0.060 |
| **Not Married (Ref)** |  |  |  |  |  |  |  |  |  |  |  |  |
| **Current infection risk** |  |  |  |  |  |  |  |  |  |  |  |  |
| **High** | 0.27 (0.20–0.36) | 2.46e-18 | 1.23 (0.53–2.85) | 0.626 | 0.30 (0.23–0.39) | 1.77e-19 | 0.86 (0.32–2.32) | 0.766 | 0.20 (0.15–0.27) | 7.80e-28 | 0.88 (0.36–2.13) | 0.776 |
| **Moderate** | 0.85 (0.53–1.38) | 0.509 | 0.73 (0.37–1.42) | 0.354 | 1.11 (0.70–1.75) | 0.655 | 0.58 (0.32–1.06) | 0.077 | 0.75 (0.44–1.26) | 0.279 | 0.52 (0.27–0.99) | 0.047 |
| **Low** | 0.92 (0.71–1.20) | 0.539 | 0.80 (0.57–1.12) | 0.193 | 0.98 (0.77–1.26) | 0.903 | 0.76 (0.56–1.02) | 0.064 | 0.98 (0.72–1.34) | 0.897 | 0.85 (0.60–1.20) | 0.361 |
| **None (Ref)** |  |  |  |  |  |  |  |  |  |  |  |  |
| **Future infection risk** |  |  |  |  |  |  |  |  |  |  |  |  |
| **High** | 0.42 (0.22–0.82) | 0.011 | 0.37 (0.15–0.89) | 0.027 | 2.44 (1.24–4.82) | 0.010 | 1.90 (0.74–4.87) | 0.183 | 0.91 (0.49–1.68) | 0.755 | 0.83 (0.39–1.74) | 0.618 |
| **Moderate** | 1.24 (0.79–1.96) | 0.352 | 1.23 (0.65–2.30) | 0.523 | 1.80 (1.17–2.77) | 0.008 | 1.81 (1.03–3.18) | 0.038 | 1.29 (0.83–2.00) | 0.256 | 1.35 (0.77–2.34) | 0.293 |
| **Low** | 1.07 (0.82–1.40) | 0.616 | 1.03 (0.73–1.46) | 0.867 | 1.48 (1.14–1.92) | 0.003 | 1.24 (0.90–1.70) | 0.182 | 1.33 (0.99–1.79) | 0.056 | 1.11 (0.80–1.55) | 0.541 |
| **None (Ref)** |  |  |  |  |  |  |  |  |  |  |  |  |

The table presents the univariate and multivariate odds ratios (OR) with 95% confidence intervals (CI) for various factors associated with ever testing for HIV, based on data from the 2018-2019, 2021, and 2022-2023 surveys. Variables include study site, gender, age group, marital status, perceived risk of infection, and future likelihood of infection. The results are presented for both univariate and multivariate models.
