# Supplementary material for: Do men face greater barriers to accessing HIV testing services than women? Might HIV self-testing be the answer? Evidence from a longitudinal survey in east Zimbabwe (2018–2023)
Source: PLOS Glob Public Health. 2026 Mar 24;6(3):e0006125. doi: 10.1371/journal.pgph.0006125 (PMC13012501; doi:10.1371/journal.pgph.0006125)
Supplement: S3 Table — The table shows the number and percentage of individuals who had knowledge of HIVST and used HIVST. (DOCX) [file pgph.0006125.s003.docx]

**S3 Table: Knowledge and Use of HIV Self-Testing (HIVST) Across the Years by Study Site, Gender, and Age Group**

| Variable | | Knowledge of HIVST | | | | Usage of HIVST | | | |
| --- | --- | --- | --- | --- | --- | --- | --- | --- | --- |
|  |  |  |  |  |  |  |  |  |  |
| Covid-19 period | | **Pre-Covid-19 (2018-2019)** | **During Covid-19 (2021)** | **Post-Covid-91 (2022-2023)** | **p-value** | **Pre-Covid-19 (2018-2019)** | **During Covid-19 (2021)** | **Post-Covid-91 (2022-2023)** | **p-value** |
|  |  | n(%) | n(%) | n (%) |  | n(%) | n(%) | n (%) |  |
| Overall | | 2656 (27.1) | 4118 (48.5) | 511 (53.1) | <0.001 | 76 (2.9) | 302 (7.3) | 521 (9.3) | <0.001 |
| Study site | Roadside settlement | 427 (25.1) | 745 (49.7) | 866 (55.8) | <0.001 | 7 (1.6) | 35 (4.7) | 52 (5.7) | <0.001 |
|  | Rural | 288 (19.2) | 377 (32.1) | 363 (29.3) | <0.001 | 3 (1.0) | 13 (3.4) | 42 (10.4) | <0.001 |
|  | Tea estate | 267 (26.2) | 470 (52.4) | 411 (43.3) | <0.001 | 13 (4.9) | 61 (13.0) | 86 (17.3) | <0.001 |
|  | Town | 849 (32.6) | 1204 (49.9) | 1342 (55.2) | <0.001 | 14 (1.6) | 67 (5.6) | 155 (10.4) | <0.001 |
|  | Forestry area | 370 (26.9 | 481 (48.9) | 775 (65.3) | <0.001 | 4 (1.1) | 41 (8.5) | 61 (7.3) | <0.001 |
|  | Urban | 455 (28.4) | 841 (55.1) | 1354 (59.9) | <0.001 | 35 (7.7) | 85 (10.1) | 125 (8.5) | <0.001 |
| Gender | Male | 1560 (38.4) | 2210 (61.6) | 2467 (59.4) | <0.001 | 33 (2.1) | 122 (5.5) | 154 (5.9) | <0.001 |
|  | Female | 1096 (19.1) | 1908 (38.9) | 2644 (48.4) | <0.001 | 43 (3.9) | 180 (9.4) | 367 (12.2) | <0.001 |
| Age group | 18-45 years | 1788 (30.7) | 2728 (55.9) | 3258 (60.0) | <0.001 | 60 (3.4) | 248 (9.1) | 439 (11.9) | <0.001 |
|  | >45 years | 458 (21.2) | 934 (38.9) | 1338 (46.6) | <0.001 | 11 (2.4) | 40 (4.3) | 57 (4.1) | <0.001 |
|  | <18 years | 410 (22.5) | 456 (37.6) | 515 (39.1) | <0.001 | 5 (1.2) | 14 (3.1) | 25 (4.6) | <0.001 |

The table presents trends in knowledge and use of HIV self-testing (HIVST) among participants from 2018-2019, 2021, and 2022-2023, disaggregated by study site, gender, and age group. The table shows the number and percentage of individuals who had knowledge of HIVST and used HIVST.
