# Supplementary material for: Do men face greater barriers to accessing HIV testing services than women? Might HIV self-testing be the answer? Evidence from a longitudinal survey in east Zimbabwe (2018–2023)
Source: PLOS Glob Public Health. 2026 Mar 24;6(3):e0006125. doi: 10.1371/journal.pgph.0006125 (PMC13012501; doi:10.1371/journal.pgph.0006125)
Supplement: S5 Table — Summary of univariate and multivariate regression analyses from generalized linear mixed-effects models evaluating factors associated with usage of HIV self-testing (HIVST). Odds ratios (ORs) and 95% confidence intervals (CIs) are reported for each variable, with p-values indicating the statistical significance of observed associations. The dependent variable was “Usage of HIVST.” Results are presented across study sites, sex, age groups, and survey rounds, with the reference categories for each variable noted. Multivariate models adjusted for potential confounders. (DOCX) [file pgph.0006125.s005.docx]

**S5 Table: Factors Associated with usage of HIV Self-Testing: Results from Generalized Linear Mixed-Effects Models**

| Variable | | Univariate | | Multivariate | |
| --- | --- | --- | --- | --- | --- |
|  |  | OR (95% CI) | *P*-value | AOR (95% CI) | *P*-value |
| Study site | Roadside settlement | 0.43 (0.27-0.69) | <0.0001 | 0.52 (0.34-0.81) | 0.003 |
|  | Rural | 0.47 (0.29-0.77) | 0.002 | 0.59 (0.37-0.95) | 0.029 |
|  | Tea estate | 1.18 (0.76-1.84) | 0.457 | 1.39 (0.91-2.12) | 1.246 |
|  | Town | 0.62 (0.40-0.96) | 0.034 | 0.67 (0.45-0.60) | 0.0588 |
|  | Forestry area | 0.58 (0.38-0.91) | 0.017 | 0.71 (0.47-1.09) | 0.115 |
|  | Urban | 1 |  | 1 |  |
| Gender | Male | 0.47 (0.41-0.54) | <0.0001 | 0.52 (0.45-0.60) | <0.0001 |
|  | Female | 1 |  | 1 |  |
| Age group | 18-45 years | 3.19 (2.31-4.40) | <0.0001 | 2.77 (2.00-3.83) | <0.0001 |
|  | >45 years | 1.38 (0.95-2.00) | 0.0876 | 1.18 (0.81-1.71) | 0.390 |
|  | <18 years | 1 |  | 1 |  |
| Survey rounds | Round 8 (2018-2019) | 2.75 (2,12-3.56) | <0.0001 | 2.66 (2.05-3.45) | <0.0001 |
|  | Round 9 (2021) | 3.55 (2.76-4.55) | <0.0001 | 3.31 (2.57-4.25) | <0.0001 |
|  | Round 7 (2022-2023) | 1 |  | 1 |  |

Summary of univariate and multivariate regression analyses from generalized linear mixed-effects models evaluating factors associated with usage of HIV self-testing (HIVST). Odds ratios (ORs) and 95% confidence intervals (CIs) are reported for each variable, with p-values indicating the statistical significance of observed associations. The dependent variable was "Usage of HIVST." Results are presented across study sites, gender, age groups, and survey rounds, with the reference categories for each variable noted. Multivariate models adjusted for potential confounders.
